# COVID-19: Preliminary Clinical Guidelines for Ophthalmology Practices

**Published:** 2020-04-06

**Authors:** Reza Gharebaghi, Jordan Desuatels, Majid Moshirfar, Maryam Parvizi, Seyed-Hashem Daryabari, Fatemeh Heidary

**Affiliations:** 1 Kish International Campus, University of Tehran, Tehran, Iran.; 2 International Virtual Ophthalmic Research Center (IVORC).; 3 The Warren Alpert Medical School, Brown University, Providence, RI, USA.; 4 HDR Research Center, Hoopes Vision, Draper, UT, USA.; 5 John A. Moran Eye Center, University of Utah School of Medicine, Salt Lake City, UT, USA.; 6 Utah Lions Eye Bank, Murray, UT, USA.; 7 Mofid Children’s Hospital, Shahid Beheshti University of Medical Sciences, Tehran, Iran.; 8 Basir Eye Health Research Center, Tehran, Iran.; 9 Taleghani Hospital, Ahvaz Jundishapur University of Medical Sciences, Ahvaz, Iran.

**Keywords:** COVID-19, Ophthalmology, Optometry, Tear, Conjunctiva, Guideline, Eye, 2019-nCoV, SARS-CoV-2

## Abstract

The zoonotic Severe Acute Respiratory Syndrome Coronavirus 2 (SARS-CoV-2) and its resultant human coronavirus disease (COVID-19) recently appeared as a global health threat that can cause severe respiratory infection and terminal respiratory distress. By the first week of April, more than 1.3 million people had been globally infected and more than 70,000 had lost their lives to this contagious virus.

Clinical manifestations occur shortly after exposure, or a few days later. There is controversy regarding the transmission of the virus through the tear and conjunctiva; however, there are reports that the ocular surface might be a potential target for COVID-19. The ease of transmission of this virus at close proximity presents a risk to eyecare workers. Several recommendations have been issued by local and national organizations to address the issue of safe ophthalmic practice during the ongoing COVID-19 pandemic. These guidelines have numerous similarities; however, subtle differences exist. The purpose of this paper was to discuss measures, with a specific focus on standard precautions, to prevent further dissemination of COVID-19 at Eye Clinics. We have proposed procedures to triage suspected cases of COVID-19, considering emergency conditions.

## INTRODUCTION

For the first time in December 2019, a Chinese ophthalmologist by the name of Dr. Li Wenliang warned of the risk of the emergence of a new and unusual type of pneumonia. On February 7, 2020, he died from COVID-19. This 33-year-old ophthalmologist was the first doctor to warn that an unusual form of respiratory infection was spreading. A few days after the beginning of the epidemic, Dr. Li Wendliang was hospitalized with a fever and cough, and he finally died. He was touted as a national hero in recognition of his sincere efforts to make the virus known to the world, and many were proud of this academic whistleblower ophthalmologist [1, 2].

On December 31, China reported an unusual outbreak of pneumonia in Wuhan, with an unknown cause, to the World Health Organization (WHO). A seafood market appeared to be the primary source of the disease, as most of those affected were in contact with the wet market. On January 7, a new coronavirus was detected by the Center for Disease Control (CDC) China named as Severe Acute Respiratory Syndrome Coronavirus 2 (SARS-CoV-2), later designated as COVID-19 by the WHO [3, 4].

The origin of this virus is unclear, although it is structurally similar to the Severe Acute Respiratory Syndrome (SARS-CoV) and the Middle East respiratory syndrome coronaviruses (MERS-CoV) [5]. Some studies have mentioned the role of bats as the main source [6], while others have mentioned the pangolin [7, 8].

**Epidemiology**

By the first week of April, 2020, more than 1.3 million people globally had been infected and more than 75,000 had lost their lives to this contagious virus. In the United States, the COVID-19 fight entered a crucial period, as the number of cases topped 360,000, with almost 10,000 deaths [9]. 

It has been estimated that the COVID-19 economic freeze could cost millions of job losses and lead to an unemployment rate of 32%, based on Federal Reserve Bank of St. Louis projections [10]. Many people have lost their jobs, the world is in a deep depression, and it is necessary to conduct multidisciplinary studies in various fields of medical and social sciences. Certainly, this has been an unexpected challenge for the world and shows that humans are still incapable of properly managing communicable diseases.

The global impact of COVID-19 is reminiscent to that of the Spanish influenza (1918-1920), which infected approximately 500 million individuals worldwide and caused the deaths of 50 to 100 million people at that time [11]. COVID-19 carried a high mortality rate [9], in contrast to the mortality rate of less than 1% from influenza therefore there is an urgent need for effective treatment [12]. To date, the death toll from COVID-19 is higher than the total registered deaths from SARS-CoV and MERS-CoV [5]. In addition, it is necessary to take preventive measures to avert future biological crises. These might include monitoring wet markets and thoroughly investigating virology laboratories. 

**Pathophysiology and Clinical Signs**

COVID-19 is an enveloped virus with a single-stranded, non-segmented, positive-sense RNA genome. The principal transmission route is through respiratory droplets [13-15]. The virus enters the body through the receptor-angiotensin converting enzyme II (ACE2), and blocking this receptor has been proposed as a potential strategy for treatment [6, 16]. Immunological evidence suggests that patients with severe COVID-19 might have cytokine storm syndrome [12], suggesting the potential of immunomodulatory agents against infection [17]. To date, there is no definitive treatment or vaccine for COVID-19, and most of the measures are based on the prevention of virus transmission. The incubation period of this viral infection is 2 to 14 days [18], with a mean of approximately 5 days, based on the present data [19].

Clinical signs and symptoms of COVID-19 include fever, dry or productive cough, fatigue, shortness of breath, myalgia, dizziness, confusion, headache, sore throat, anorexia, hemoptysis, rhinorrhea, chest pain, diarrhea, nausea and vomiting, and anosmia [3, 4, 20, 21]. Remarkably, the mortality rate associated with COVID-19 is significantly higher compared to the most recent seasonal influenza [22] and may be exacerbated in the elderly and those with comorbidities. Also, the mortality rate is higher in centers with a shortage of intensive care facilities. In comparison with the characteristics of H1N1 patients, COVID-19 cases are more likely to reveal non-productive cough with constitutional symptoms, such as fatigue, gastrointestinal symptoms, and prevalence in the aging subgroup. Additionally, radiological features are more frequently presented as ground-glass opacities in COVID-19 cases in comparison with H1N1 [23]. On the other hand, we believe that since COVID-19 patients require a ventilator for longer durations, this will indirectly limit the available supply and place a pressing demand for additional ventilators that is unexpectedly above anything we have witnessed before.

There are no reports comparing the rate of infection among healthcare workers. However, with rising numbers and the influx of patients to the emergency room, the most frontline healthcare workers, paramedics, nurses, and doctors in all specialties, including emergency physicians, anesthesiologists, pulmonologists, and respiratory therapists are at imminent risk.

Figures from China revealed that more than 3,300 healthcare workers were infected as of early March, 2020, whereas at the end of February, 2020, at least 22 workers had died. In Italy, almost 20% of responding healthcare personnel are currently infected, and some have died [24]. Reports have shown that 43 healthcare workers have died in Iran [25].

## COVID-19 AND THE EYE

A close distance between the patient and the ophthalmologist is a risk factor for COVID-19 transmission. The CDC recommends social distancing, whereby people should be at least 2 meters apart to avoid transmission of the virus. Any contact longer than a few minutes of exposure is considered prolonged [26]. However, in ophthalmic examination, the distance between the patient and the ophthalmologist is as close as 20 cm in most circumstances, and the time required for a full assessment including taking history, checking visual acuity, measuring intraocular pressure, waiting for pupil dilation, etc., is more than half an hour. In ocular examination, contact with the patient is inevitable. 

As stated in the introduction, the first report was from an ophthalmologist who reported a COVID-19 patient and died of COVID-19 himself [1]. Studies on other betacoronaviruses have shown that they are likely to be recovered through tears [27]. Even though the risk may be quite small, studies have shown tears as a contaminated ocular secretion. In a prospective interventional study, researchers conducted a reverse transcription-polymerase chain reaction (RT-PCR) on the tears and conjunctival secretion of 30 patients with COVID-19 pneumonia, and found that one patient with conjunctivitis had positive results for RT-PCR. This suggests that the virus might infect the conjunctiva and cause conjunctivitis [28]. In another study, researchers reported conjunctival congestion in 9 of 1099 patients with COVID-19 [29]. In a study from Singapore, 64 tear samples were collected from 17 patients with COVID-19 between days 3 to 20 of the onset of the primary symptom, but viral culture and RT-PCR showed negative results. The researchers, therefore, concluded that the risk of transmission through tears is low. Although this was the first report to assess the possibility of COVID-19 transmission through tears, it had some limitations such as a small number of subjects and technical limitations [30]. Although studies have shown a low probability of COVID-19 transmission, it is still possible to transmit the virus due to the patient's long stay in the Eye Clinic, short distance between the ophthalmologist and the patient, and the possibility of transmission via respiratory droplets.

In a case series including 38 patients with COVID-19, 12 patients had ocular manifestations, such as conjunctival congestion, epiphora, or chemosis, and these signs frequently appeared in cases with more severe systemic manifestations. Results revealed a low prevalence of nucleotides in conjunctival specimens of patients with COVID-19, whereas only one patient presented with conjunctivitis as the first symptom [31], showing that the rate of conjunctivitis was quite low.

Feline and murine models have shown that coronaviruses can affect the eye. The ocular implications of human corona virus infections have not been extensively studied. However, coronaviruses are generally known to cause numerous ocular infections in animals. Clinical findings such as conjunctivitis, retinitis, anterior uveitis, and optic neuritis have been acknowledged in animal models [32]. 

Studies show that COVID-19 is transmitted by asymptomatic individuals, making it difficult to control [33]. However, it should be noted that inadequate screening methods in most geographical regions might lead to underdiagnosis, especially in patients with less serious illness. 

Furthermore, a medical officer who visited Wuhan wore an N95 mask but without protecting his eyes reported that eye redness can even appear before respiratory symptoms [34].

**Clinical Guidelines and Legal Issues**

Local and international ophthalmology authorities have published recommendations at the International Council of Ophthalmology (ICO) website [35], although the creation and implementation of evidence-based clinical guidelines specific for COVID-19 at the Eye Clinic, for the assessment, investigation, and management of high-risk patients is necessary.

There is an example of comprehensive recommendations by the American Academy of Ophthalmology (AAO) [36] and a well-designed study from Hong Kong [37]. However, it is the responsibility of global organizations to review daily evidence and provide more comprehensive and uniform guidelines. This paper aimed to provide an evidence-based approach, although this may not be applicable to all centers, and is not a proven medico-legal recommendation. Subsequent information about the nature of COVID-19 may change these recommendations. It should be noted that some countries have limited facilities and medical resources. Healthcare resources should be wisely allocated, and it is the responsibility of healthcare policymakers to ensure fairness in the allocation process.

Another important point is the legal and ethical debate on nosocomial COVID-19 infection. Ethics committees must rapidly make revisions to existing protocols. Ethics experts must respond to the challenge if a patient is hospitalized for ophthalmic surgical interventions, but becomes infected with COVID-19 and eventually dies, or decide what to do if someone complains of irreversible complications because of delayed surgery due to COVID-19 prevention.

Today, a new chapter in the history of infectious diseases is being written [38], and a multidisciplinary approach should focus on different aspects of this challenging health crisis.

## GENERAL RECOMMENDATIONS

**A. Triage Protocol**

In any case, it is imperative to compare the risk of COVID-19 transmission with the risk to the patient if not treated, and this risk-benefit analysis should be based on clinical judgment. Preventive protective measures for patients and clinic staff should begin outside the clinic. If feasible, a standardized patient screening protocol, comprising a concise but comprehensive review of constitutional signs and symptoms of COVID-19 including fever, dry or productive cough, fatigue, shortness of breath, myalgia, dizziness, confusion, headache, sore throat, anorexia, hemoptysis, rhinorrhea, chest pain, diarrhea, nausea and vomiting, anosmia, presence of conjunctivitis [3, 4, 20, 21, 28, 31], recent travel, and contact with sick individuals should be accomplished over the phone prior to the scheduled patient visit. Patients should also be specifically queried about recent positive, negative, or pending COVID-19 test results obtained for themselves and close proximity contacts. The ocular chief complaint should also be gathered remotely by phone or electronic records and subjected to the chief provider’s clinical judgment to determine visit urgency. 

The aforementioned screening protocol will hopefully reduce unnecessary in-person visits and allow for the stratification of patients into groups based on low or high COVID-19 risk and low or high ophthalmologic acuity. Patients with low ophthalmologic acuity may be appropriate for either referral to a later appointment date or tele-ophthalmology services. 

The demonstrated efficacy of tele-ophthalmology in rural settings shows the potential for extrapolating this technique to other practice settings [39]. However, evidence for the use of electronic ophthalmology in the elderly is scarce and may be further limited for patients with visual impairments [40]. 

If there is access to patients' contact information, they could be notified that their non-emergency surgery or visit has been postponed. They should also be reminded of COVID-19 symptoms and contact local health authorities or a family doctor if appropriate. 

Table 1 presents hypothetical measures in Eye Clinics during the COVID-19 pandemic. At the triage desk, the patient's body temperature (**T**) is measured. Questions regarding clinical signs and symptoms (**S**) of COVID-19 include whether there is fever, dry or productive cough, fatigue, shortness of breath, myalgia, dizziness, confusion, headache, sore-throat, anorexia, hemoptysis, rhinorrhea, chest pain, diarrhea, nausea and vomiting, and anosmia, conjunctivitis, history of contact with COVID-19 patients (**CO**), or travel history (**T**) to involved geographical areas. The patient would be considered **COST-positive** if any of these factors were present. These high-risk patients should be directed to the Red Room, which is a separate room, for the examination of suspicious cases. Standard precautions should be exaggerated in this room, including the use of personal protective equipment (PPE) and meticulous disinfection of surfaces.

In COST-negative cases, it is important to check whether the patient's eye condition is an emergency. If there is no emergency, such as the need for refraction or routine check-up, the patient should be asked to go home until the COVID-19 crisis is resolved. However, if the patient's condition is an emergency and they are COST-negative, intervention should be made based on clinical judgment. 

**Table 1:** Risk assessment of cases at the triage desk of Eye Clinics during the COVID-19 pandemic.

High-Risk Patients (COST-positive) *
Presence of Potential COVID-19 Signs and Symptoms (**S**) **
Contact with COVID-19 patients (**CO**)
High Body Temperature (**T**)
Travel history to high-risk areas (**T**)

* Anyone who is not in the above list should be considered low risk. For urgent eye problems in low-risk patients, personal protective equipment (PPE) need only be goggles and gloves, gown, special mask for the ophthalmologist, and regular face mask for the patients. Note: This screening recommendation is based on current evidence. There is no priority placed on any item. ** Clinical signs and symptoms of COVID-19 include fever, dry or productive cough, fatigue, shortness of breath, myalgia, dizziness, confusion, headache, sore-throat, anorexia, hemoptysis, rhinorrhea, chest pain, diarrhea, nausea and vomiting, anosmia, and conjunctivitis [3, 4, 20, 21, 28, 31].

**Table 2:** Levels of urgency based on considerations for the management of cases. Cases should be categorized into groups by local authorities based on clinical judgment and available resources [42].

Levels of Urgency
• **Emergency** cases should be performed within 24 hours **OR** as soon as possible to preserve sight and/or life.
• **Urgent** cases should be performed within 1 week, considering the availability of resources.
• **Semi-urgent** cases should be performed within 1-2 months, considering the availability of resources.
• **Non-urgent** cases should be deferred for at least 2-3 months or until improved availability of local operating room resources.

**Figure 1:**
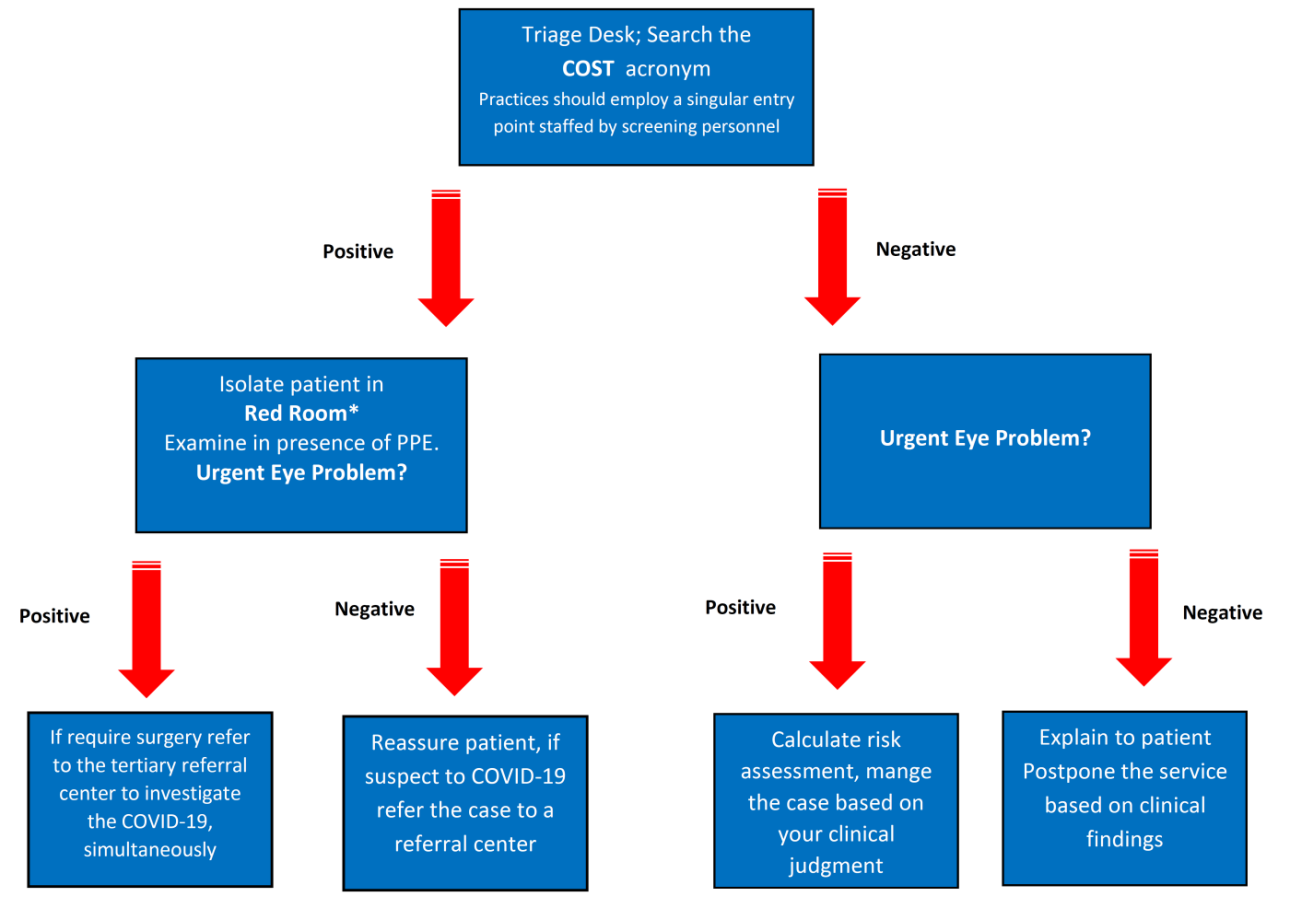
Decision tree in the Eye Clinic during the COVID-19 pandemic.

COST: CO, contact with COVID-19 patient; S, presence of potential signs and symptoms; F, fever; T, travel history to high-risk areas.

* The Red Room would be equivalent to an Operating Room (OR) with a negative pressure environment, which is ideal for reducing dissemination of the virus beyond the OR [45]. The minimal composition of the PPE set in the Red Room should be:

- Respiratory protection (N95 mask or equivalent for healthcare workers, 3 Ply face masks for patients)
- Hand protection gloves (preferably double gloving in high-risk cases)
- Body protection long-sleeved water-resistant gown
- Eye protection goggles or face shield

**Figure 2:**
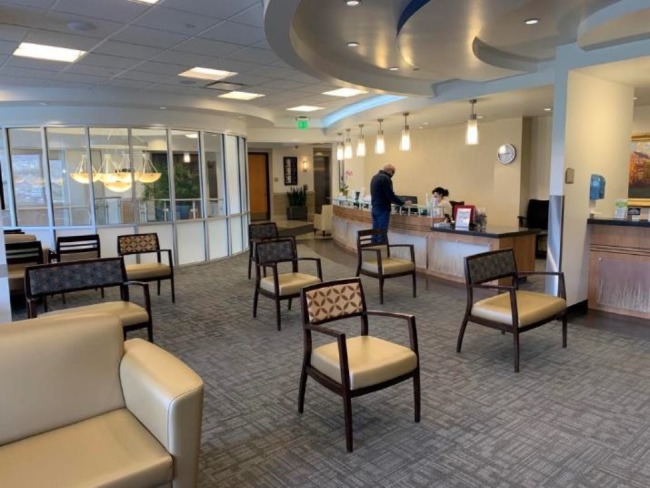
Patient waiting areas should adhere to proper social distancing protocols. Chairs should be spaced accordingly. Photo Courtesy of Majid Moshirfar, MD FACS.

**Figure 3:**
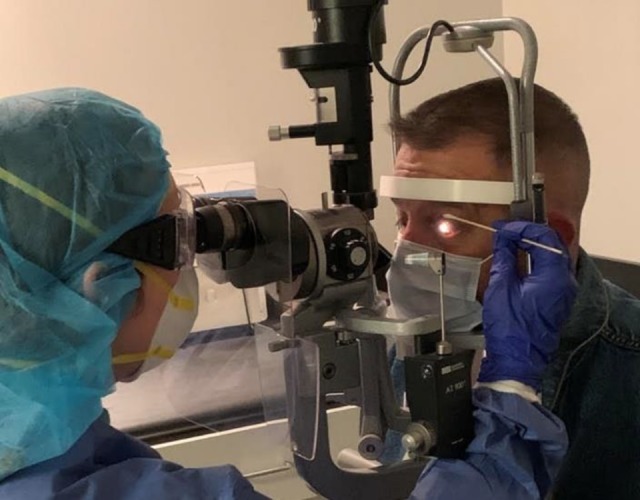
Suggested slit lamp examination of a low-risk patient in clinic. Note lid manipulation should not be accomplished by hand. The physician is shown wearing the suggested attire of a surgical gown, gloves, N95 mask, surgical bonnet, and protective glasses. Photo Courtesy of Majid Moshirfar, MD FACS.

**Figure 4:**
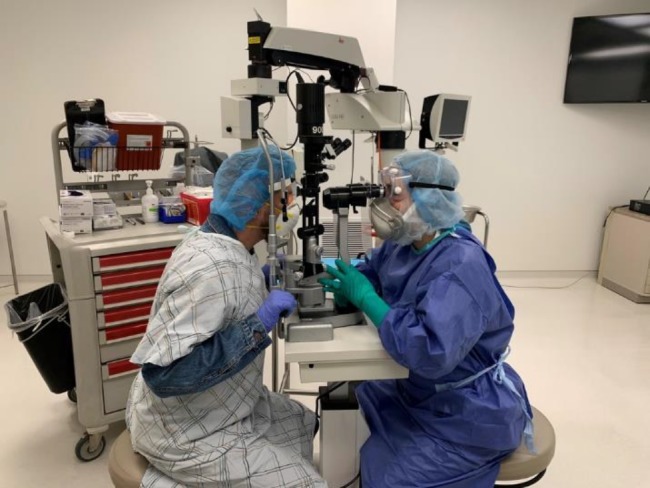
Suggested slit lamp examination set up for a high-risk patient. Note the operating room setting with negative pressure ventilation capabilities. The examiner is wearing extensive respiratory protection, goggles, facial covering, hair covering, surgical gown, and gloves. Photo Courtesy of Majid Moshirfar, MD FACS.

**Figure 5:**
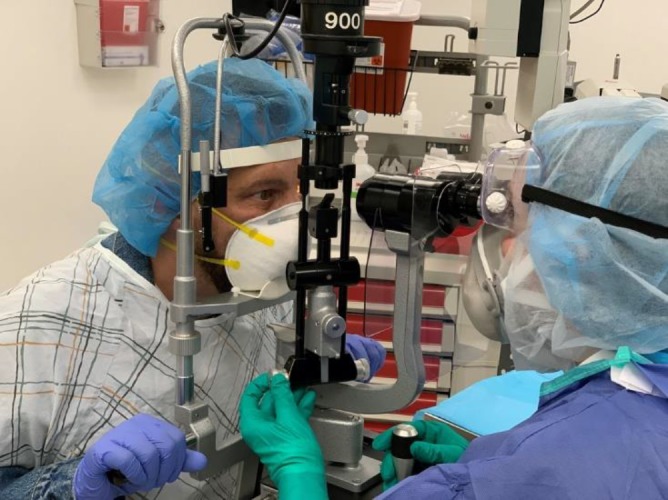
Close up viewpoint demonstrating slit lamp examination of a high-risk patient. A two clear plastic barriers can be seen attached to the binocular eye piece and lens. Photo Courtesy of Majid Moshirfar, MD FACS.

**Figure 6:**
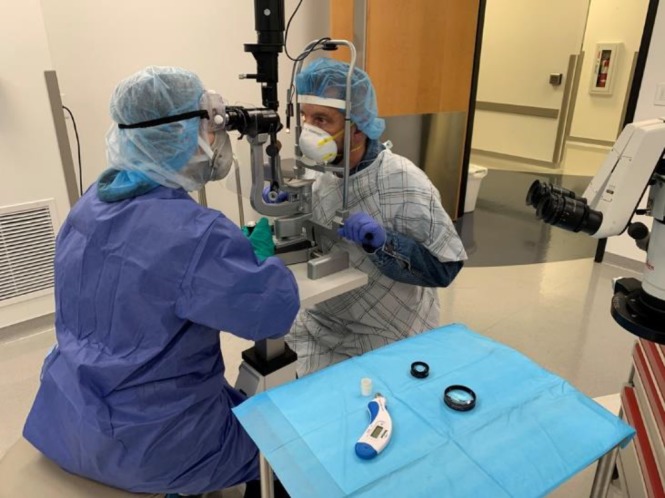
Glass lenses and Tonopen arranged on a disposable surgical underpad. Photo Courtesy of Majid Moshirfar, MD FACS.

**B. Necessity of Personal Protection**

PPE is a significant component, but only one part of the system protects staff and other patients from cross-infection. All clinical staff, including screening personnel, should wear PPE in congruence with regional guidelines, availability, and appropriateness to the symptomatology of the patient being treated. At a minimum, all staff should be outfitted with a respiratory barrier having N95 or greater filtration capacity and gloves. Staff who examine patients in very close proximity such as with a slit lamp, or who operate on, or otherwise instrument the eye, should use ocular protection, gloves, and the examiner should only touch the patients’ eyes using a disposable cotton-tip applicator. Patients at higher risk may also be outfitted with similar PPE to reduce spread of the virus through droplets or contact. 

PPE is necessary to reduce the risk of exposure, and may include gloves, goggles, face shields, water resistance gowns, and respiratory protective equipment, especially in the setting of the Red Room. Medical staff in the ophthalmology clinic, ward, and operation room should have standard insurance coverage. 

It is clear that the re-use of masks should be avoided, as these might pose a risk to healthcare workers.

Some guidelines advocate double gloving in high-risk cases [41]. The health protection of healthcare workers is a top priority. Just as in an airplane, it is recommended that one apply an oxygen mask themself, before helping others. Therefore, healthcare workers have to be healthy to provide a good service to their patients. Hence, maintaining the health of medical staff and providing protective equipment for them is a priority.

**C. Work Flow**

If tele-ophthalmology or phone-based consultation is not appropriate for a given scenario, in-person visits should be carried out at extended intervals, perhaps of 1 to 1.5 hours, to avoid patient overlap. Additional and redundant screening should take place at the point of care for patients requiring in-person consultation or treatment at a sanctioned healthcare facility. Practices should consider the implementation of screening personal and high-visibility signage outside the entrance of their facility to recapitulate the screening questions mentioned above, as well as take direct temperature measurements to screen for active fever. Exterior signage should also make patients aware of appropriate social distancing strategies, such as the maintenance of 2 meter spacing between persons. These additional screening steps may capture patients who were missed by phone screening or developed symptoms between the time of phone screening and presentation. We need to make sure that after initial screening at the entry door to the healthcare facility, and before entering the waiting area, all patients have a face mask or be provided with one. In view of the enormous shortage of surgical masks, the wearing of even a low-grade non-surgical face mask should be enforced at all times. No one should be allowed to walk around the facility without a face mask.

Standard procedures should be developed to categorize patients according to their COVID-19 risk and clinical requirements. All patients should maintain a proper distance of two meters when possible and should not be allowed to congregate in waiting rooms or other shared spaces. Reduce the number of seats in the waiting room Always keep a few empty exam rooms so the elderly and immunocompromised patients can be escorted directly from the reception area, bypassing the waiting area. The waiting area should be preferably designated for low-risk patients only. Reduce the number of personnel by rotating them and space the diagnostic devices and computer monitors at least 6 meters from one another. Body temperature of personnel and the checking for symptoms related to infection should be assessed every day. Once COVID-19 screening becomes more available and cost effective, with a 15-minute turnaround time, one may consider changing the personnel every several days to screen for asymptomatic infection. To the best of the clinic’s abilities, patients should be brought directly into exam or procedural rooms that are as highly spaced as possible, and properly sanitized between patients. High-risk patients should be considered for placement in an operating room with negative pressure ventilation, referred to as a Red Room in this paper. We suggest placing one slit-lamp using a proper distance in one of the operating rooms in the facility. Discriminating between an emergency and non-emergency condition depends entirely on the patient's condition and the clinician’s judgment. Monocular patients should be considered as special cases. 

Table 2 presents the levels of urgency in ocular oncology based on considerations for the management of cases during the COVID-19 pandemic [42]. To step up infection control measures at the Eye Clinic, in partnership with major societies, the AAO has collated a list of urgent and emergency procedures generally accomplished in operating rooms, which may be considered as the reference list [36]. 

For example, patients with immature cataract, epiretinal membrane, or those needing refractive surgery could be scheduled later. However, in cases such as endophthalmitis, prompt action should be taken. In ocular pathologies, malignancies should be prioritized, and those with a higher degree of malignancy should be given more attention. In particular, attention should be paid to situations that lead to death or disability. Manipulating a patient's respiratory system, such as nasal endoscopy, should be avoided as much as possible.

**D. Medical Instruments and the Clinical Environment**

The clinical environment, including instruments such as occluders, prisms, trial frames, trial lenses, and goniolens should be disinfected properly based on the official recommendations.

A disposal applanation tonometer head is preferred. Although 70% alcohol seems to be sufficient for disinfection of the tonometer, it has not been shown effective against adenoviruses. Direct contact between the tonometer and the cornea may increase the risk of cross-infection. There is limited data available regarding the transmission of HIV, hepatitis B, hepatitis C, and prion diseases through the use of tonometers [43]. However, as studies have shown that diseases can be transmitted through close contact, the use of a non-contact tonometer for measurement of the intraocular pressure is recommended whenever possible. Of course, the air-puff tonometer that causes tears to form microparticles is under question.

Specific equipment requiring close proximity use, such as the slit lamp, should be appropriately adapted. Although of questionable efficacy, it has been suggested that plastic breath shields be placed between the slit lamp examiner and subject. Although there are no standard recommendations, the use of a slit lamp breath shield is recommended to reduce the transmission of droplets. 

This outbreak is an opportunity for the development of new directions in e-health. Perhaps another future direction to consider as we enter a world with unpredictable seasonality of COVID-19 infection, and the ever-present possibility of novel pandemics, is the development of remotely controlled slit lamps that can be controlled from a location separate from the patient. This highlights the urgency of the evolution of robotics and tele-medicine in ophthalmology.

The surfaces of cell phones, keyboards, and face masks, which are frequently used, are among the most contaminated areas. 

Washing hands with soap and water before entering the examination room is an important practice. Instructing patients on proper hand washing, using brochures, posters, etc., is recommended. Proper ventilation should be available in the office. All equipment should be sanitized between each patient’s use by appropriately trained staff. Diagnostic devices are also subject to 2-meter distancing parameters and should be appropriately moved or unplugged to ensure proper adherence. 

The coronavirus family can persist for up to 6 days on some surfaces based on some evidence [44]. It is very important to clean the floor, appliances, and areas that the patient is in contact with (such as a slit-lamp handle and chin rest). After each examination, the equipment used, especially the patient's contact point, should be disinfected by trained staff. Each Eye Clinic should have an affordable, effective, and low-cost method of cleaning surfaces and equipment based on local protocols. One person should regularly monitor the implementation of these protocols, acting as the COVID-19 Quality Control Officer. 

Gloves should be replaced after each examination. Do not exchange cash in the office. The patient's file and receipt should not be given to him, and the process should be carried out electronically. 

The examination room should have proper ventilation. Limiting conversation in the waiting and examination rooms should be considered. 

**E. Surgical Considerations**

It is generally advisable not to perform any intervention in the clinic, especially for patients with positive COST conditions, and instead refer them to tertiary referral centers (Figure 1). In the present health crisis, the postponement of elective surgery and routine examinations is recommended. 

Patients must complete a COVID-19 consent form prior to surgery. If the patient needs surgery, a screening test to rule out COVID-19 prior to surgery is recommended. In the operating room, surgical procedures should be performed remotely and expeditiously. However, precision should not be sacrificed. It is advisable not to perform general anesthesia as much as possible. In both the clinic and the operating room, the health of healthcare workers is very important and should be protected meticulously. Their body temperature should be checked regularly, and they should complete a symptom checklist at regular intervals. Figure 2-6 show the general consideration to visit patients at eye clinic in different scenarios.

**F. Other Considerations**

Many patients use contact lenses and cannot function without them. We believe rigorous contact lens hygiene should be emphasized, but the use of contact lenses should be optional, unless someone has viral conjunctivitis or an ocular manifestation of COVID-19.

It is necessary to pause non-COVID-19-related research and to convert ophthalmology teaching to online training, if possible. Ophthalmology promotion exams can also be postponed until further notice. As residents in governmental departments are at the frontline against COVID-19, economic and psychological incentive mechanisms for them and their families should be defined. Last but not least, mental health is as important as fighting the virus. The anxiety epidemic is growing much larger than the COVID-19 epidemic itself. It should be noted that people are naturally anxious about this situation. 
